# Craniofacial development in patients of Tessier No.0 cleft with a bifid nose using 3D computed tomography

**DOI:** 10.3389/fped.2022.979345

**Published:** 2022-08-24

**Authors:** Xin Wang, Huan Wang, Jianjun You, Ruobing Zheng, Yihao Xu, Fei Fan

**Affiliations:** Department of Rhinoplasty, Plastic Surgery Hospital, Chinese Academy of Medical Sciences and Peking Union Medical College, Beijing, China

**Keywords:** Tessier No.0 cleft with a bifid nose, cephalometries, skull base, development, analysis

## Abstract

**Objective:**

Considerable studies have focused mainly on the facial deformity of Tessier No.0 cleft with a bifid nose, but the deformity of the skull is not well understood. Therefore, our study aimed to explore the evolution of cranial dysmorphology and the chronology of Tessier No.0 cleft with a bifid nose, by three-dimensional measurements.

**Methods:**

Ninety-six non-surgical patients and computed tomographic scans were included (Tessier No.0 cleft with a bifid nose, *n* = 48; controls, *n* = 48) and divided into five age subgroups. Craniofacial cephalometric measurements were analyzed by Mimics software.

**Results:**

The widening of nasal bone was the most remarkable and persistent from 2 years old appropriately. The overall cranial base length in patients compared with controls increased 11.8% (*p* < 0.01) on average. The middle and posterior cranial fossa increasing accounted for most of this change. The cranial base angles also showed increased obviously. By analyzing the linear of the nasopharynx and respiratory tract, it was found that its development did not affect respiration.

**Conclusions:**

The cranial base deformity of Tessier No.0 cleft with a bifid nose consists of the whole skull base and particularly the middle and posterior cranial base length increase. At the same time, there may be late closure of the spheno-occipital synchondrosis and sella displacement. We believe this study is unique in providing valuable data for elucidating the pathological and morphological abnormalities of skull base development in Tessier No.0 cleft with a bifid nose.

## Introduction

Tessier No.0 cleft with a bifid nose is characterized by its flat dorsum, short nose, separated alar cartilages, and separation of ascending maxillary processes, which is a rare congenital anomaly (1, 2). The markedly bifid nose is a concave groove along the entire length of the dorsum. The most common are orbital hypertelorism and midline cleft lip (2). Less common symptoms are eye defects, anal deformities, and intracranial anomalies, such as frontonasal encephalocele and intellectual disability (3, 4). Our former research was focused on the facial features of Tessier No.0 cleft with a bifid nose to analyze the deformities of the bifid nose and found the defects between the patients and ordinary people (5). However, facial malformation in Tessier No.0 cleft with a bifid nose is consistent with cranial base development in time? At present, there is no such study at home and abroad.

Three-dimensional measurement methods have gained a better understanding of the structural features of these facial deformities recently (6, 7). Exact measurements are necessary to detect deformities and to focus further efforts on skull development. Some literature has pointed out that facial features may be related to the development of the skull base (8, 9). Thus, the purpose of this study was to try to explore the above question and define the craniofacial influences which change with age, by objectively analyzing craniofacial developments.

## Method

This is a longitudinal study performed following the institutional human investigation committee and all methods were performed following the relevant guidelines and regulations. Computed tomographic scans were collected from all Tessier No.0 clefts with a bifid nose without a history of any previous surgical intervention from 2012 to 2022. Accompanied by median cleft lip or palate, intracranial anomalies and other craniofacial syndromes were excluded in our analysis. We collected each patient's data, such as sex, age, and associated anomalies. All patients were divided into five subgroups based on their ages: 0 to 6 months, 6 months to 2 years, 2 to 6 years, 6 to 18 years, and over 18 years old. We also adopted a group of normal people as the control group with age- and sex-matched.

**Figure 1:**
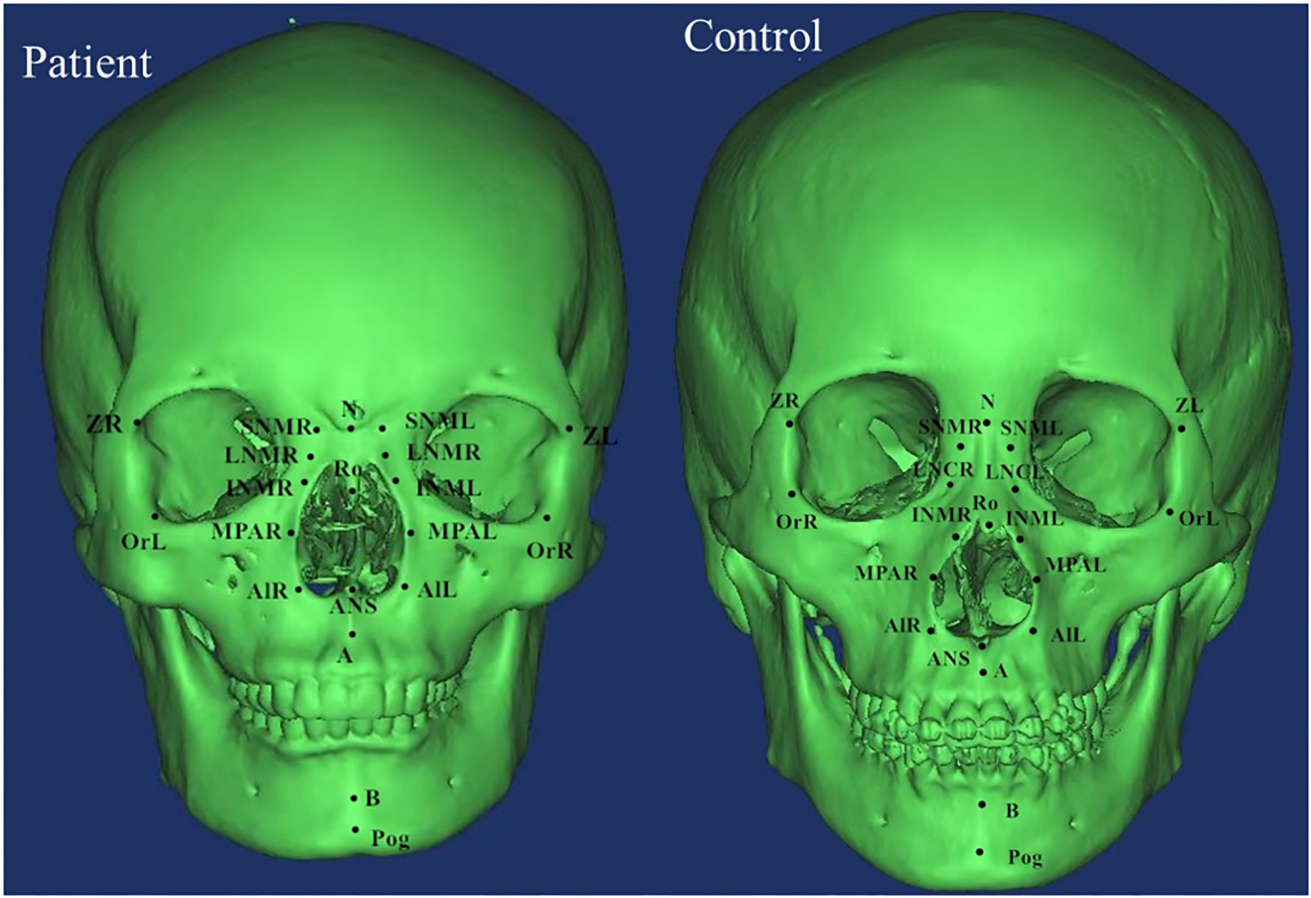
The anatomical landmarks were shown on the computed tomographic scans (coronal scan).

**Figure 2:**
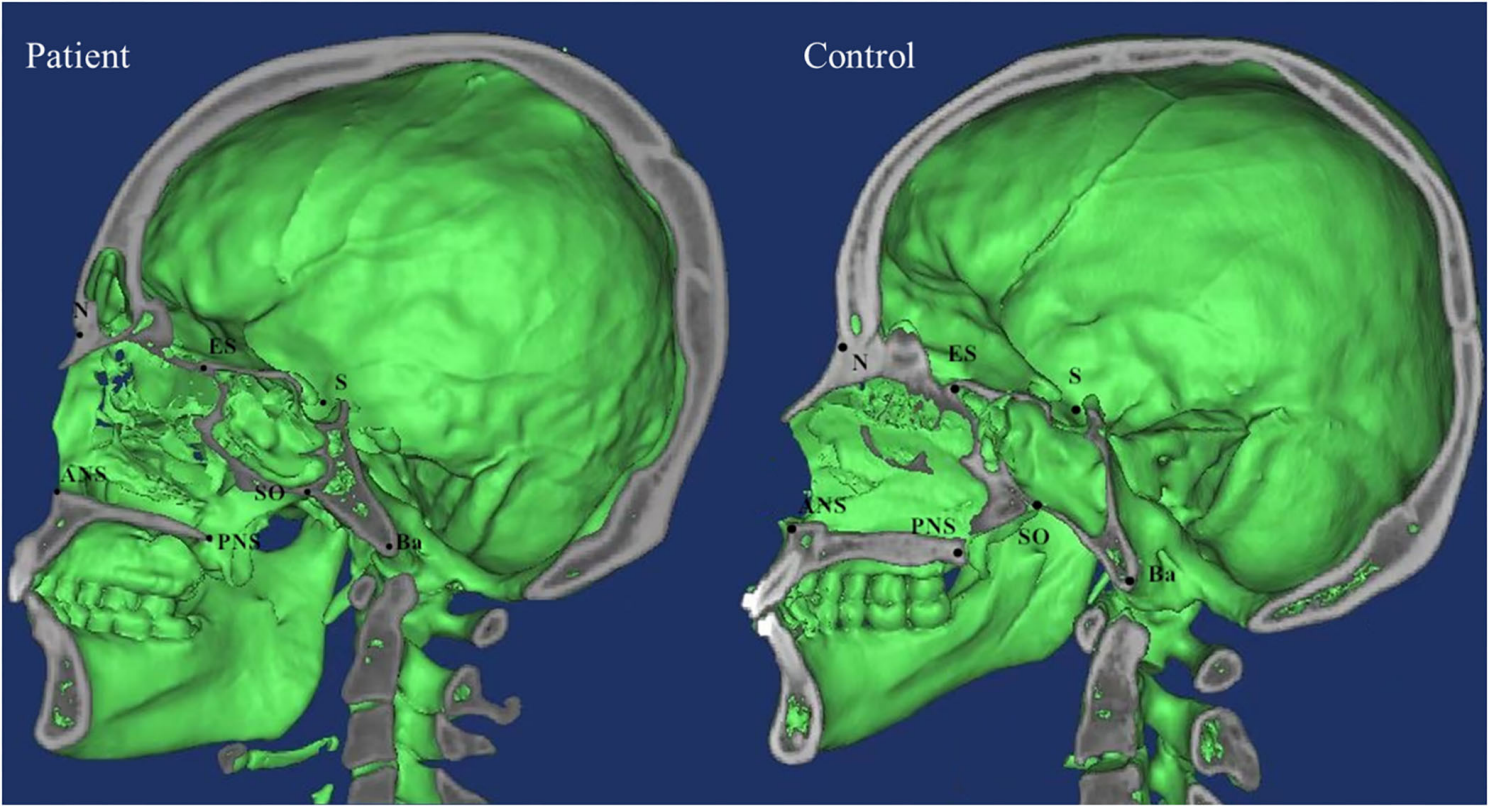
The anatomical landmarks were shown on the computed tomographic scans (sagittal scan).

DICOM data were analyzed using Mimics software which was used to determine the marking points and measure the distances and angles between the left and right marking points, and lines. Measurements in both patients and control groups were taken independently by two examiners (XW and RZ) and verified by two additional observers (HW and YX) based on the Cephalometric Reference System by Swennen et al. and other literatures (6, 10, 11). Significant landmarks included zygomaticofrontal suture (Z), orbitale (Or), superior nasomaxillary suture (SNM), lateral nasal center point (LNC), middle point of lateral piriform aperture (MPA), alare (Al), nasion (N), basion (Ba), sella (S), anterior nasal spine (ANS), posterior nasal spine (PNS), pogonion (Pog), spheno-occipital synchondrosis (SO), and ethmo-sphenoid (ES). The definitions of landmarks, cephalometric distances, and angles were summarized in Supplementary Table 1 and landmarks were shown in Figures 1, 2.

All statistical analyses were performed using statistical software (SPSS, version 24.0; IBM Corp., Armonk, New York, USA) and figures were made with Prism 8.0 software. We collected as many patients as possible and we did not carry out a power analysis to assess the sample size. A test of normality was produced using the Shapiro–Wilk test. A *t*-test was used for the comparison of continuous variables. Independent *t*-tests and one-way analysis of variance (ANOVA) were used to examine the differences between the two groups. A *p*-value <0.05 was considered statistically significant. Pearson correlation coefficients were used to evaluate the correlation among the measured indicators with statistical differences and *r* > 0.6 was identified as a strong correlation.

## Result

### Demographic data

A total of 96 computed tomographic scans were included (Tessier No.0 cleft with a bifid nose patient, *n* = 48; control, *n* = 48). The median age of the patients and controls were 10.78 and 11.14 years, respectively. The five age subgroups consisted of the following: 0 to 6 months (*n* = 2), 6 months to 2 years (*n* = 2), 2 to 6 years (*n* = 18), 6 to 18 years (*n* = 13), and over 18 years old (*n* = 13). Both groups consist of 23 male and 25 female patients. Table 1 showed the summary of the characteristics of the patients.

**Table 1:** Demographic information of Tessier No.0 cleft with a bifid nose and controls.

**Age subgroup**	**Tessier No.0 cleft with a bifid nose**	**Controls**	***P*-value**
0–0.5 y			
Mean age	0.41 ± 0.07	0.40 ± 0.09	0.92
No. (male/female)	2 (1/1)	2 (1/1)	
0.5–2 y			
Mean age	1.65 ± 0.21	1.60 ± 0.28	0.86
No. (male/female)	2 (2/0)	2 (1/0)	
2–6 y			
Mean age	4.47 ± 1.08	4.27 ± 0.94	0.57
No. (male/female)	18 (6/12)	18 (6/12)	
6–18 y			
Mean age	11.69 ± 3.32	12.27 ± 3.94	0.76
No. (male/female)	13 (7/6)	13 (7/6)	
≥18 y			
Mean age	26.62 ± 3.77	26.27 ± 3.49	0.35
No. (male/female)	13 (7/6)	13 (7/6)	
Total			
Mean age	10.78 ± 7.94	11.14 ± 8.12	0.30
No. (male/female)	48 (23/25)	48 (23/25)	

### Linear measurements (overall)

From the Coronal view of craniofacial, we found that Tessier No.0 cleft with bifid nose patients had increased orbital width (OrR–OrL) and zygoma length (ZL-ZR), but not significantly. However, the development of nasal bone was the most remarkable and persistent. The width of upper nasal bone (SNML-SNMR), width of middle nasal bone (LNCL-LNCR), width of lower end of nasal bone (INML-INMR), middle nasal width (MPAL-MPAR), and Alar point distance (AlL-AlR) were wider than controls (*p* < 0.05).

In general, compared with the control group, the distance between the middle and posterior skull bases increased in the Tessier No.0 cleft with a bifid nose group. The overall cranial base length (N-Ba) increased 11.8% in patients compared with controls (*p* < 0.01). To the measurement of posterior cranial fossa, S-Ba, and SO-Ba were increased significantly, except S-SO was decreased. The middle cranial fossa related to SO-ES and S-ES, which the latter one was increased significantly and it is the most influential contributor to the increased cranial base length. The N-ES was decreased by 10.40% and S-N did not change significantly, compared with controls, which mainly showed the anterior cranial fossa length. Specifically, with ANS as the reference point, the upper anterior facial height (N-ANS) and Ba-NAS were decreased by 8.2% (*p* = 0.03) and 15.21% (*p* < 0.01), but S-ANS and ANS-PNS showed no change. As for the PNS, the upper posterior facial height (S-PNS), Ba-PNS, and ES-PNS showed no change.

### Angular measurements

The SNA and SNB did not change significantly, but ANB did. The facial convexity angle (NA-PA) was increased by 4.02 degrees in patients compared with controls. The cranial base angle in the neurocranium (intracranial surface) of the skull (N-S-BA) was decreased significantly. The angles N-S-SO and S-SO-BA increased 7.73 degrees (*p* < 0.01) and 23.24 degrees (*p* < 0.01), respectively, whereas the angle Ba-S-ES increased 25.15 degrees (*p* < 0.01) compared with controls. The above analysis was summarized in Table 2.

**Table 2:** Cranial base linear and angular measurements in the full age range of Tessier No.0 cleft with a bifid nose compared to controls.

**Index**	**Full age**		
	**Patients**	**Controls**	***P*-value**
**Coronal view of face linear measurements**			
ZL-ZR	93.27 ± 10.42	89.64 ± 9.16	0.07
OrL-OrR	84.39 ± 10.28	81.06 ± 11.95	0.15
SNML-SNMR	14.78 ± 3.71	9.30 ± 1.58	<0.01**
LNCL-LNCR	16.84 ± 4.11	12.56 ± 1.68	<0.01**
INML-INMR	17.79 ± 4.68	15.58 ± 2.03	0.01*
MPAL-MPAR	25.36 ± 5.10	21.14 ± 3.98	<0.01**
AlL-AlR	23.56 ± 4.30	21.27 ± 3.80	0.01*
**Sagittal view of face**			
**linear measurements**			
N-Ro	15.37 ± 5.14	18.63 ± 3.60	<0.01**
ANS-PNS	46.13 ± 7.82	46.00 ± 6.98	0.93
N-ANS	39.07 ± 6.62	42.57 ± 8.46	0.03*
N-PNS	67.80 ± 9.16	63.65 ± 10.76	0.04*
**Cranial base inner linear measurements**			
N-Ba	104.89 ± 12.24	93.81 ± 12.54	<0.01**
S-Ba	48.42 ± 8.34	38.89 ± 7.50	<0.01**
S-N	59.56 ± 8.72	61.11 ± 7.78	0.36
S-SO	19.19 ± 3.46	21.46 ± 3.65	<0.01**
S-ES	23.36 ± 3.77	21.32 ± 3.00	<0.01**
SO-Ba	28.50 ± 6.74	21.46 ± 3.65	<0.01**
SO-ES	33.36 ± 5.37	33.54 ± 4.48	0.86
N-ES	37.06 ± 5.80	41.36 ± 6.59	<0.01**
**Cranial base external linear measurements**			
Ba-ANS	64.59 ± 7.83	76.18 ± 10.24	<0.01**
Ba-PNS	47.15 ± 9.71	44.86 ± 8.56	0.10
S-ANS	66.95 ± 8.43	70.44 ± 3.88	0.05
S-PNS	39.05 ± 7.29	39.05 ± 6.31	0.99
ES-PNS	39.07 ± 7.09	38.29 ± 7.62	0.60
**Angular measurements**			
SNA	86.20 ± 7.55	85.17 ± 6.00	0.46
SNB	80.64 ± 7.67	80.87 ± 6.80	0.87
ANB	6.16 ± 3.00	4.65 ± 1.77	<0.01**
NA-PA	13.88 ± 6.71	9.86 ± 4.35	<0.01**
N-S-Ba	118.06 ± 13.39	124.18 ± 8.24	<0.01**
N-S-SO	93.47 ± 10.68	101.20 ± 3.40	<0.01**
S-SO-Ba	102.91 ± 15.97	126.15 ± 4.48	<0.01**
N-SO-Ba	171.79 ± 7.07	172.45 ± 4.47	0.59
Ba-S-ES	154.40 ± 8.40	129.25 ± 5.70	<0.01**

*P <0.05; **P <0.01.

### Age subgroup analysis

Before 6 months of age, only a few measurements appeared to change. The distance N-Ba showed a persistent increase with a significant difference (*p* < 0.01). The N-Ro, S-Ba, Ba-ANS, and ES-PNS had statistical differences compared with controls initially and then grew close to normal, between 6 months and 2 years. The angles of SNA and SNB decreased statistical difference only in this stage. The angles S-SO-Ba and Ba-S-ES have always increased compared with controls through all subgroups. The N-S-Ba was decreased by 18.24 degrees before 6 months (*p* < 0.01) but normalized from 6 months to 2 years, yet repeatedly decreased compared to controls later on.

From 6 months to 2 years of age, SO-Ba began to increase significantly and has been different since then. No other distance and angles had statistically significant.

From age 2–6 years, the development of nasal bones began to show some differences.

The distance of SNML-SNMR, LNCL-LNCR, MPAL-MPAR, and AlL-AlR was all increased by more than 20% (*p* < 0.01) except the INML-INMR which increased between 6 and 18 ages. The ANS-PNS and N-PNS also had statistical differences only in this stage. S-SO of Cranial base inner linear measurement began as shortened by 13%. External linear measurements Ba-ANS and S-ANS were also shortened. The relatively stable angles of ANB, NA-PA, and N-S-SO showed significantly different and remained greater until over 18 years old.

**Figure 3:**
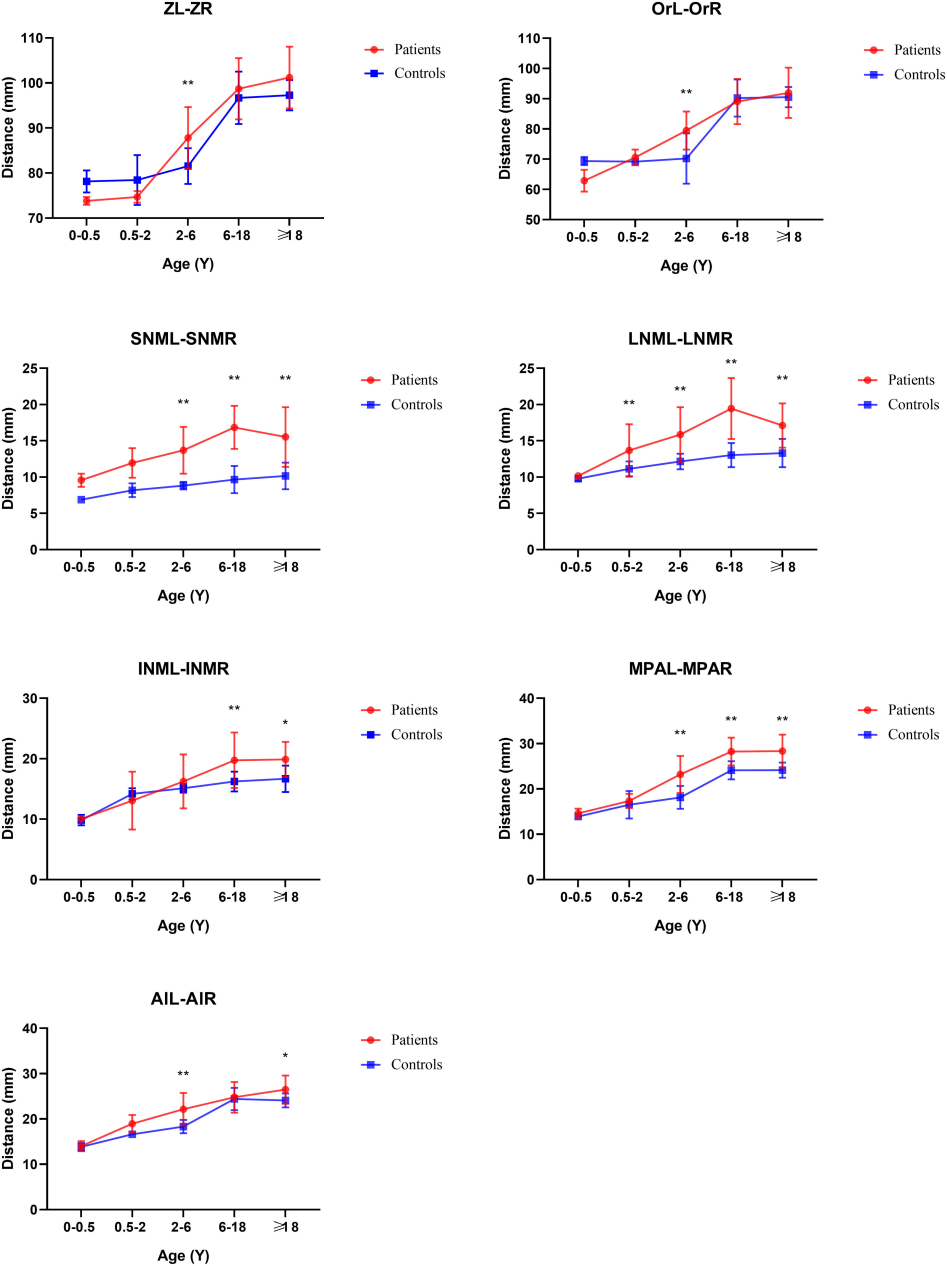
Coronal view of face linear measurements by subgroup analysis. *P < 0.05; **P < 0.01.

**Figure 4:**
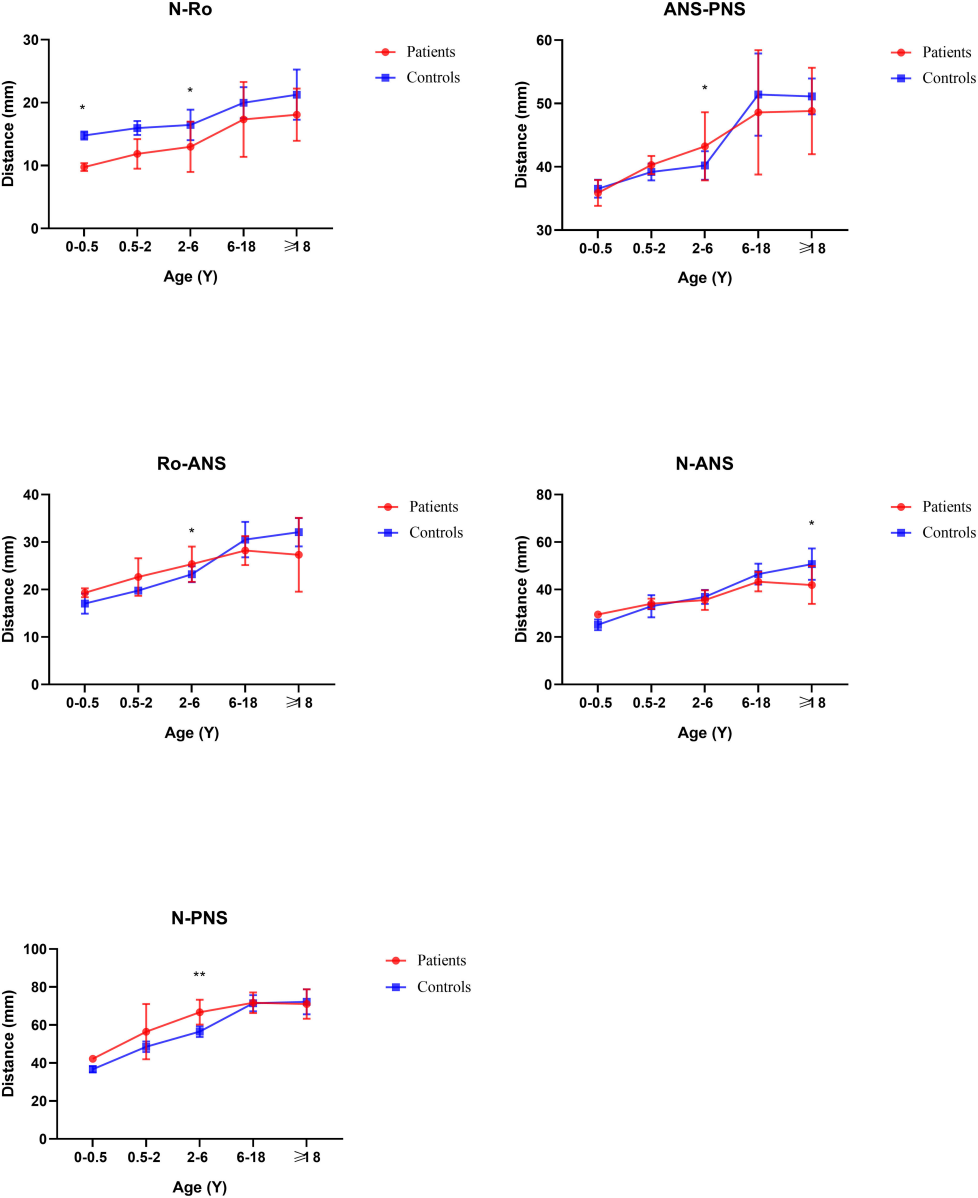
Sagittal view of face linear measurements by subgroup analysis. *P < 0.05; **P < 0.01.

**Figure 5:**
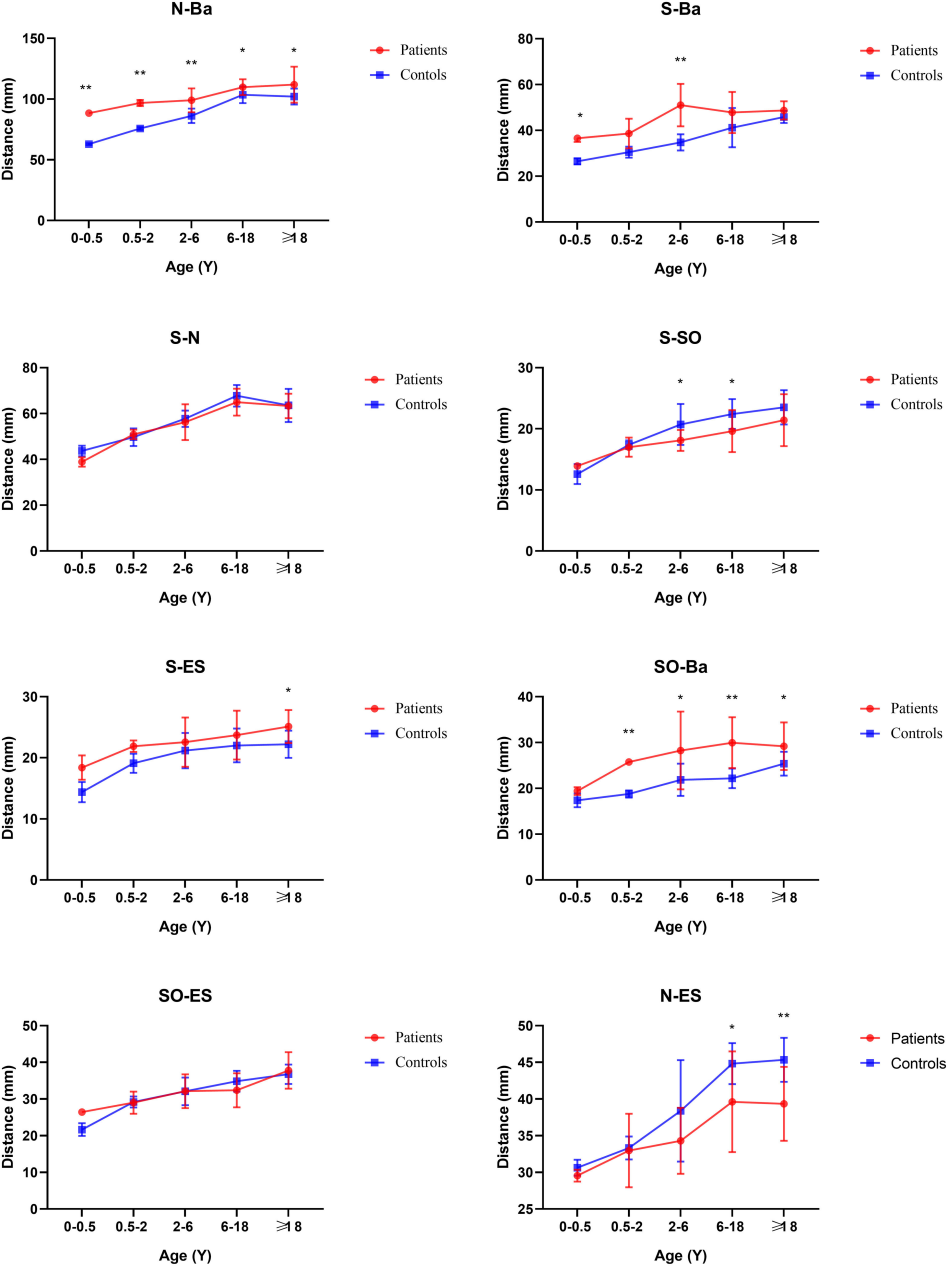
Cranial base inner linear measurements by subgroup analysis. *P < 0.05; **P < 0.01.

**Figure 6:**
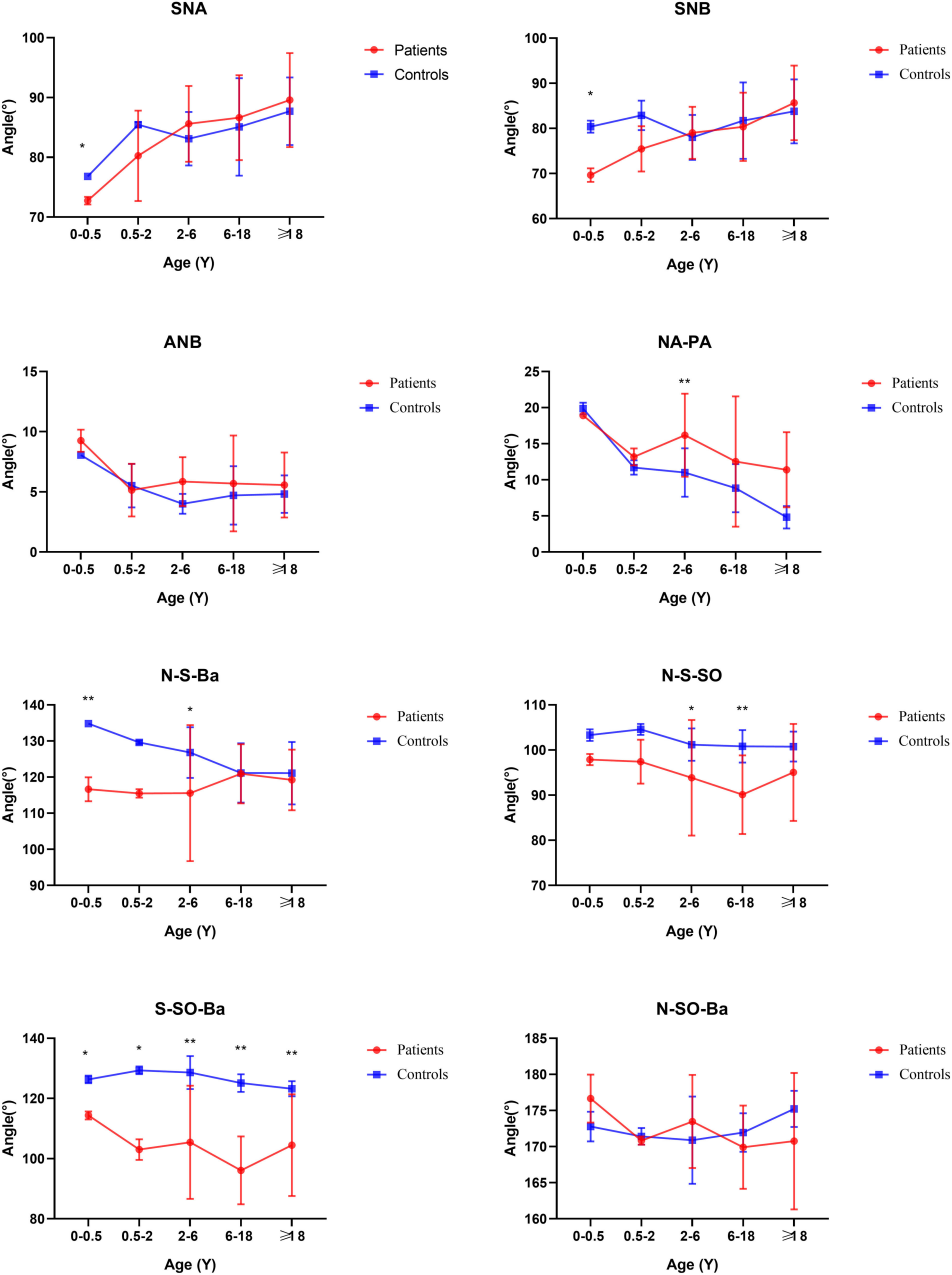
Cranial base external linear measurements by subgroup analysis. *P < 0.05; **P < 0.01.

**Figure 7:**
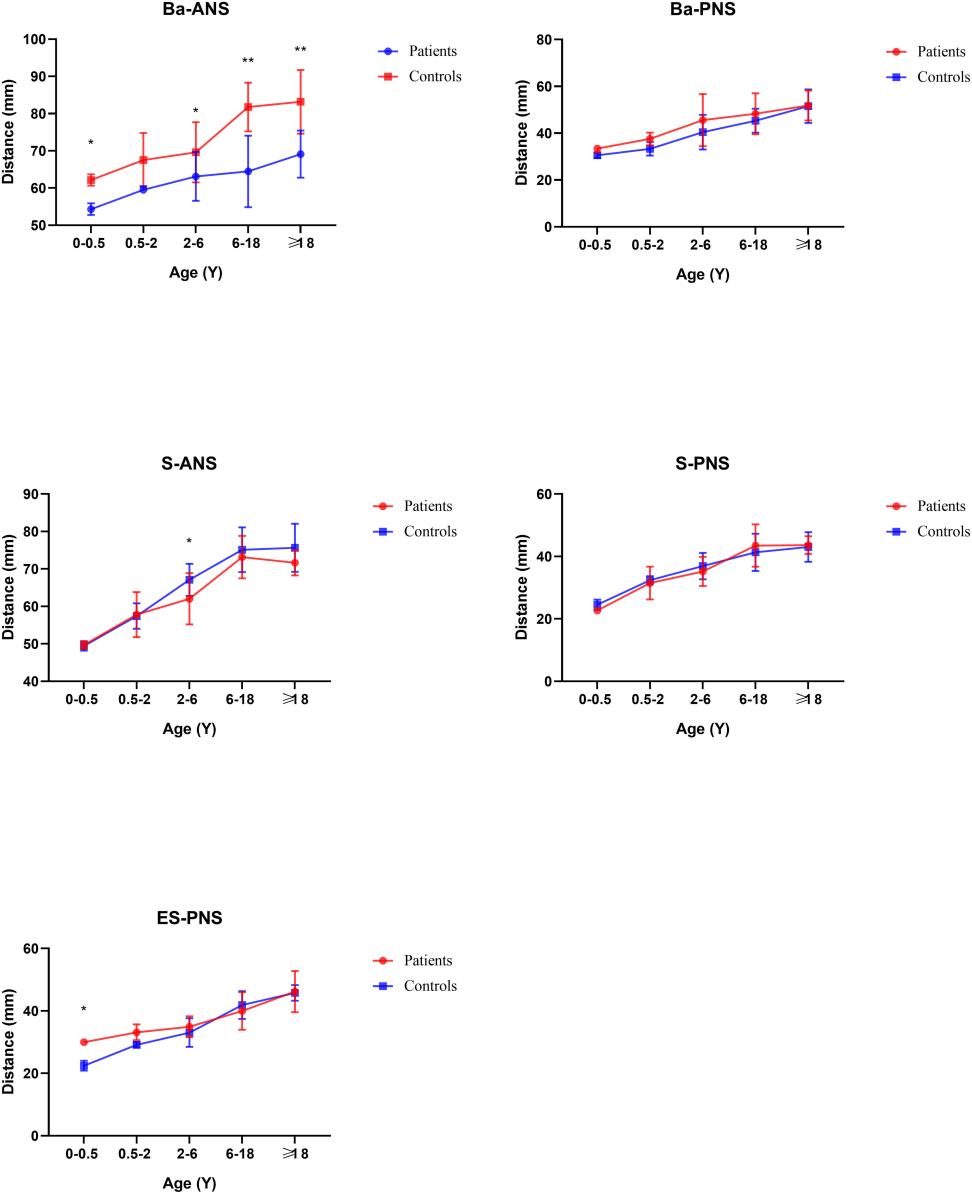
Angular measurements by subgroup analysis. *P < 0.05; **P < 0.01.

From age 6–18 years and over 18 years old, S-N, SO-ES, and N-SO-Ba did not yield statistical significance in the age subgroups and overall analysis. Besides the changed measurements mentioned above, N-ANS, and S-ES began statistical significance. The subgroup analysis of linear and angular measurements is reported in Supplementary Table 2; Figures 3–7.

### Correlation analysis

**Figure 8:**
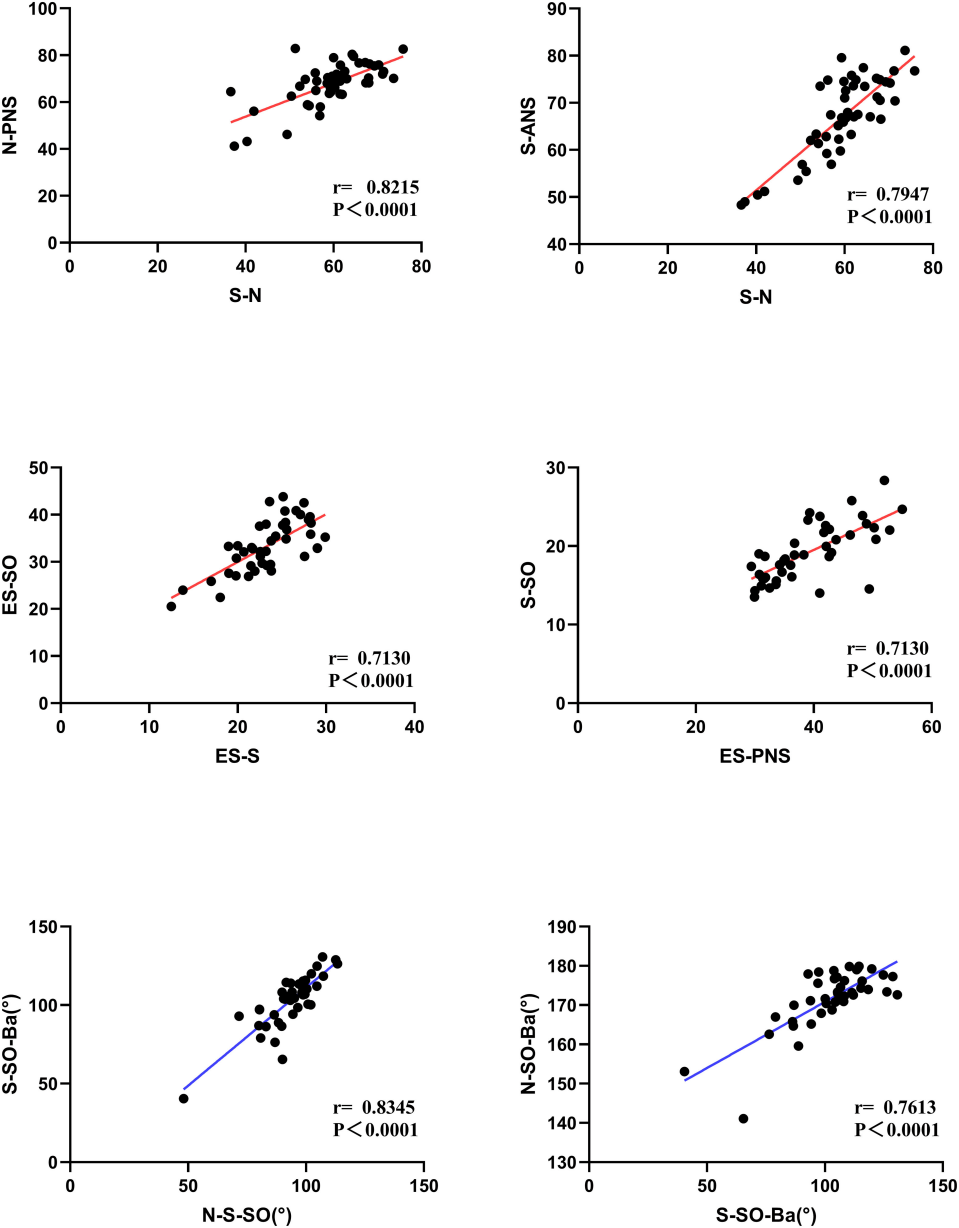
The correlation analysis of distances and angles.

The correlation analysis of distances and angles is shown in Figure 8. The lengths of S-N were more related to N-PNS (*r* = 0.8215) and S-ANS (*r* = 0.7967) in patients. Moreover, there were positive significant correlations of the length ES-S to ES-SO (*r* = 0.7130) and ES-PNS to the length of S-SO (*r* = 0.7130). There are strong correlations between the angles of S-SO-Ba to N-S-SO (*r* = 0.8345) and N-SO-Ba (*r* = 0.7613).

## Discussion

Tessier No.0 cleft with a bifid nose is rare and the etiology is still unclear with two main types. One believed that the failure of fusion of mesoderm and ectoderm led to cleft and many craniofacial clefts were related to gene expression (12, 13). The other was that the occurrence of facial fissures was related to the lack of a potential ectoderm-penetrating fusion line (14). In general, its main performance was the malformation involving a nasal supporting structure or soft tissue. Since some studies believed that the occurrence of the facial cleft was related to some genes related to craniofacial malformation such as IRF6, ALX1, ALX3, and ADH1C (12, 15). Our study was to explore whether there was skull deformity in Tessier No.0 cleft with a bifid nose and how the abnormal morphology of the skull base evolved.

Based on our data (SNML-SNMR, LNCL-LNCR, INML-INMR, AlL-AlR, and N-Ro), the flattening and widening of the nasal bone are one of the earliest and most evident regions of developmental deformities in Tessier No.0 cleft with a bifid nose. Furthermore, these deformities were consistent over time and progressive in age as measured subsequently. That is why the patients' nasal dorsum is collapsed and flat, and the nose is short, which is in agreement with their clinical manifestations. In addition, Orbitale width was increased significantly, only in 2–6 years of age, which just showed that not all patients had the performance of orbital hypertelorism and it is consistent with the description in the literature (1).

For the base of the skull, there are two key anatomical structures. One is sella and the other is spheno-occipital synchondrosis. At birth, the skull base is mostly composed of cartilage, and then gradually ossified (16). Therefore, abnormal ossification may lead to an uncommon position and size of sella. The significantly shortened distance between S and SO suggested the anteroposterior dimension of the sella was decreased. Furthermore, the significantly decreased angle of N-S-BA and N-S-SO, indicated the sella was moved forward. It is reported that premature fusion of the SO could lead to shortening of the skull base and affect the angle of the skull base (17). According to our measurement results, the fusion may occur as early as 2 years old. But this was remarkably earlier than controls whose SO's closure time was approximately 11–18 years of age by McGrath et al. (18). This manifestation has also been reported in Apert and Crouzon syndromes (7, 11).

Compared with controls, the whole cranial base length was increased from birth, which was different from Apert and Crouzon syndromes with shortened length. The main increase in length was the posterior cranial fossa and middle cranial fossa, but the anterior cranial fossa length did not change noticeably. At the 6-month point, there was abnormal growth in medio-posterior in the skull. A hypothesis is the late closure of anterior and posterior fontanels and the cranial base moves upward as compensation, which is consistent with the increased cranial linear measurements and the upward sella, mentioned above. The cranial base external linear measurements indicate the increased distance between Ba and PNS, but not surprisingly. In patterns of palatal plane descent follow, the Ba-PNS is longer to Bachmayer et al. (19). It reflects the cause of the lengthening of the whole posterior skull base.

From the sagittal view of face linear measurements, Ba-PNS, Ba-ANS, and ANS-PNS have not remarkably changed. They formed a radial line at the bottom of the nasal airway, so it could be seen that they did not affect the volume of the airway, which was in agreement with previous studies (20). For the measurement of maxillary morphology such as ANS-PNS, N-ANS, and SNA, they did not show a difference. Moreover, SNB and ANB also have no change, which further explains that the airway mostly was not obstructed. This is different from Treacher-Collins syndrome with decreased SNB and increased ANB (21). To summarize, the volume of the nasopharynx cavity of the Tessier No.0 cleft with a bifid nose did not decrease, and the possibility of respiratory dysfunction was also lower.

The limitation of this study was there are few patients with Tessier No.0 cleft with a bifid nose before 2 years old, and subgroups have a large age range between them (i.e., more than 18 years of age). The inclusion criterion of this study was that the patients must not have undergone any previous surgery. Thus, unoperated patients are very rare. In this situation, we believe this study is unique in providing valuable data for elucidating the pathological and morphological abnormalities of skull base development in Tessier No.0 cleft with a bifid nose.

## Conclusion

The cranial base deformity of Tessier No.0 cleft with a bifid nose consists of the whole skull base and particularly the middle and posterior cranial base length increase. At the same time, there may be late closure of the spheno-occipital synchondrosis and sella displacement. Age subgroup analysis showed that the cranial base deformity was likely to occur at 2 years old and mainly affects the development of the posterior cranial fossa. By analyzing the linear of the nasopharynx and respiratory tract, it was found that its development did not affect respiration.

## Data availability statement

The original contributions presented in the study are included in the article/Supplementary material, further inquiries can be directed to the corresponding author/s.

## Ethics statement

The studies involving human participants were reviewed and approved by Plastic Surgery Hospital, Chinese Academy of Medical Sciences, and Peking Union Medical College. Written informed consent to participate in this study was provided by the participants' legal guardian/next of kin. Written informed consent was obtained from the individual(s), and minor(s)' legal guardian/next of kin, for the publication of any potentially identifiable images or data included in this article.

## Author contributions

XW and HW designed and performed the experiments. XW and RZ collected the data. HW and JY analyzed and verified the data. XW searched the literature and wrote the manuscript. FF revised the manuscript. All authors contributed to the article and approved the submitted version.

## Conflict of interest

The authors declare that the research was conducted in the absence of any commercial or financial relationships that could be construed as a potential conflict of interest.

## Publisher's note

All claims expressed in this article are solely those of the authors and do not necessarily represent those of their affiliated organizations, or those of the publisher, the editors and the reviewers. Any product that may be evaluated in this article, or claim that may be made by its manufacturer, is not guaranteed or endorsed by the publisher.
