# Supplementary material for: Craniofacial development in patients of Tessier No.0 cleft with a bifid nose using 3D computed tomography
Source: Front Pediatr. 2022 Aug 24;10:979345. doi: 10.3389/fped.2022.979345 (PMC9449123; doi:10.3389/fped.2022.979345)
Supplement: Supplementary file 1 [file Table_1.DOCX]

Supplement Table 3. Age Subgroup Analysis in Tessier No.0 Cleft with a Bifid Nose Compared with Controls over Time

| Index | 0-0.5 y | | | 0.5-2 y | | | 2-6 y | | |
| --- | --- | --- | --- | --- | --- | --- | --- | --- | --- |
|  | Patiens | Controls | P value | Patiens | Controls | P value | Patiens | Controls | P value |
| Coronal view of face linear measurements |  |  |  |  |  |  |  |  |  |
| ZL-ZR | 73.82±0.61 | 78.14±1.74 | 0.14 | 74.69±1.28 | 78.45±5.53 | 0.45 | 87.81±6.83 | 81.55±3.97 | ＜0.01^**^ |
| OrL-OrR | 62.88±2.56 | 69.38±0.95 | 0.14 | 70.58±2.58 | 69.20±4.15 | 0.46 | 79.48±6.30 | 70.20±8.34 | ＜0.01^**^ |
| SNML-SNMR | 9.57±0.64 | 6.89±0.02 | 0.05 | 12.00±2.04 | 8.19±0.95 | 0.14 | 13.70±3.52 | 8.83±0.52 | ＜0.01^**^ |
| LNCL-LNCR | 10.16±0.16 | 9.78±0.24 | 0.31 | 13.68±3.61 | 11.14±1.01 | 0.44 | 15.86±3.77 | 12.15±1.07 | 0.01^*^ |
| INML-INMR | 10.03±0.29 | 9.89±0.66 | 0.86 | 13.07±4.78 | 14.20±0.92 | 0.78 | 16.25±4.47 | 15.10±0.72 | 0.30 |
| MPAL-MPAR | 14.62±0.28 | 13.89±0.46 | 0.48 | 17.36±1.54 | 16.52±3.02 | 0.76 | 23.21±4.09 | 18.13±2.51 | ＜0.01^**^ |
| AlL-AlR | 14.00±0.80 | 13.84±0.61 | 0.89 | 19.00±1.93 | 16.62±0.71 | 0.25 | 22.16±3.59 | 18.32±1.46 | ＜0.01^**^ |
| Sagittal view of face  linear measurements |  |  |  |  |  |  |  |  |  |
| N-Ro | 9.77±0.45 | 14.78±0.46 | 0.02^*^ | 11.87±2.35 | 15.97±1.12 | 0.16 | 12.99±4.00 | 16.46±2.42 | 0.01^*^ |
| ANS-PNS | 35.88±1.45 | 36.54±1.01 | 0.74 | 40.27±1.76 | 39.19±1.32 | 0.33 | 43.23±4.28 | 40.20±2.25 | 0.04^*^ |
| N-ANS | 29.49±0.51 | 25.14±1.60 | 0.12 | 34.01±2.18 | 32.98±4.66 | 0.80 | 35.56±4.14 | 36.87±2.93 | 0.28 |
| N-PNS | 42.2±1.01 | 36.76±1.22 | 0.08 | 56.48±9.53 | 48.55±2.73 | 0.53 | 66.74±6.51 | 56.50±2.78 | ＜0.01^**^ |
| Cranial base inner linear measurements |  |  |  |  |  |  |  |  |  |
| N-Ba | 88.43±2.01 | 62.92±0.52 | ＜0.01^**^ | 96.78±2.49 | 75.78±1.15 | ＜0.01^**^ | 99.02±7.76 | 86.23±5.92 | ＜0.01^**^ |
| S-Ba | 36.54±1.11 | 26.49±0.95 | 0.02^*^ | 38.62±6.48 | 30.50±2.40 | 0.24 | 51.03±9.28 | 34.74±3.53 | ＜0.01^**^ |
| S-N | 38.88±1.45 | 43.69±1.63 | 0.16 | 50.89±2.06 | 49.68±3.81 | 0.73 | 56.2±7.80 | 57.74±5.54 | 0.45 |
| S-SO | 13.90±0.40 | 12.60±1.15 | 0.40 | 17.00±1.56 | 17.40±0.71 | 0.77 | 18.1±1.71 | 20.71±3.34 | 0.01^*^ |
| S-ES | 18.40±1.40 | 14.40±1.17 | 0.16 | 21.90±0.94 | 19.10±1.56 | 0.16 | 22.56±4.03 | 21.18±2.89 | 0.24 |
| SO-Ba | 19.40±0.60 | 17.38±1.06 | 0.24 | 25.77±0.50 | 18.77±0.78 | ＜0.01^**^ | 28.26±8.50 | 21.85±3.52 | 0.01^*^ |
| SO-ES | 26.44±0.57 | 21.65±1.22 | 0.07 | 29.00±3.01 | 29.18±1.49 | 0.95 | 32.13±4.62 | 32.09±3.77 | 0.98 |
| N-ES | 29.55±1.17 | 30.64±0.89 | 0.89 | 32.98±5.01 | 33.32±1.57 | 0.93 | 34.30±4.50 | 38.83±6.92 | 0.05 |
| Cranial base external linear measurements |  |  |  |  |  |  |  |  |  |
| Ba-ANS | 54.32±1.11 | 62.16±1.07 | 0.04^*^ | 59.54±0.98 | 67.52±7.31 | 0.27 | 63.12±6.54 | 69.61±8.09 | 0.01^*^ |
| Ba-PNS | 33.43±1.11 | 30.50±0.74 | 0.16 | 37.54±2.71 | 33.31±2.87 | 0.27 | 43.87±11.13 | 40.43±7.37 | 0.11 |
| S-ANS | 49.70±0.73 | 49.43±0.89 | 0.84 | 57.80±6.00 | 57.42±3.40 | 0.95 | 62.04±6.89 | 67.09±4.27 | 0.01^*^ |
| S-PNS | 22.66±0.66 | 24.51±1.17 | 0.30 | 31.48±5.23 | 32.38±1.29 | 0.84 | 35.18±4.70 | 36.92±4.23 | 0.25 |
| ES-PNS | 29.98±0.02 | 22.45±1.11 | 0.02^*^ | 33.15±2.54 | 29.14±0.84 | 0.17 | 34.94±3.36 | 33.04±4.56 | 0.16 |
| Angular measurements |  |  |  |  |  |  |  |  |  |
| SNA | 72.75±0.45 | 77.32±0.88 | 0.04^*^ | 80.26±7.56 | 85.47±0.42 | 0.07 | 85.60±6.35 | 83.11±4.47 | 0.18 |
| SNB | 69.65±1.05 | 80.38±0.95 | 0.02^*^ | 75.47±5.03 | 82.88±3.27 | 0.22 | 79.02±5.77 | 78.01±4.98 | 0.58 |
| ANB | 9.25±0.65 | 8.05±0.15 | 0.21 | 5.15±2.18 | 5.52±1.80 | 0.87 | 5.85±2.51 | 4.01±0.82 | 0.07 |
| NA-PA | 18.90±0.10 | 19.87±0.57 | 0.24 | 13.17±1.17 | 11.70±0.99 | 0.31 | 16.18±5.74 | 11.01±3.36 | ＜0.01^**^ |
| N-S-Ba | 116.60±0.20 | 134.84±0.61 | ＜0.01^**^ | 115.89±6.63 | 129.62±0.47 | 0.06 | 115.57±8.82 | 126.80±7.01 | 0.03^*^ |
| N-S-SO | 97.88±0.88 | 103.30±0.90 | 0.05 | 97.42±4.87 | 104.58±1.21 | 0.18 | 93.97±12.80 | 101.20±3.58 | 0.03^*^ |
| S-SO-Ba | 114.37±0.94 | 126.32±0.89 | 0.01^*^ | 103.01±3.44 | 129.38±1.34 | 0.01^*^ | 105.44±8.99 | 128.61±5.48 | ＜0.01^**^ |
| N-SO-Ba | 176.65±2.35 | 172.78±1.45 | 0.30 | 170.83±0.57 | 171.46±1.22 | 0.58 | 173.61±6.45 | 170.89±6.05 | 0.22 |
| Ba-S-ES | 158.10±1.30 | 141.82±1.50 | 0.02^*^ | 150.16±1.03 | 131.85±0.69 | ＜0.01^**^ | 153.28±8.29 | 130.22±5.59 | ＜0.01^**^ |

| 6-18 y | | | ≥18 y | | |
| --- | --- | --- | --- | --- | --- |
| Patients | Controls | P value | Patients | Controls | P Value |
|  |  |  |  |  |  |
| 98.72±6.80 | 96.70±5.82 | 0.42 | 101.21±6.85 | 97.29±3.37 | 0.08 |
| 89.06±7.48 | 90.24±6.14 | 0.67 | 91.94±8.32 | 90.53±3.34 | 0.58 |
| 16.84±2.99 | 9.65±1.88 | ＜0.01^**^ | 15.52±4.12 | 10.15±1.83 | ＜0.01^**^ |
| 19.45±4.21 | 13.03±1.67 | ＜0.01^**^ | 17.11±3.05 | 13.30±1.95 | ＜0.01^**^ |
| 19.74±4.59 | 16.23±1.64 | 0.02^*^ | 19.89±2.40 | 16.68±2.19 | ＜0.01^**^ |
| 28.24±3.01 | 24.12±2.00 | ＜0.01^**^ | 28.35±3.63 | 24.15±1.66 | ＜0.01^**^ |
| 24.78±3.38 | 24.00±2.44 | 0.74 | 26.47±3.12 | 24.09±1.54 | 0.02^*^ |
|  |  |  |  |  |  |
| 17.33±5.94 | 19.99±2.47 | 0.15 | 18.09±4.14 | 21.27±3.99 | 0.06 |
| 48.60±9.81 | 51.40±6.49 | 0.40 | 48.82±6.82 | 51.11±2.83 | 0.27 |
| 43.31±4.10 | 46.48±4.40 | 0.07 | 41.92±7.99 | 50.70±6.59 | 0.01^*^ |
| 71.75±5.42 | 71.46±4.23 | 0.89 | 71.02±7.76 | 71.19±6.59 | 0.68 |
|  |  |  |  |  |  |
| 109.78±8.26 | 103.53±6.85 | 0.03^*^ | 111.90±11.40 | 102.10±6.56 | 0.04^*^ |
| 47.83±8.97 | 41.20±8.57 | 0.07 | 48.70±4.00 | 45.86±2.66 | 0.06 |
| 64.96±5.88 | 67.75±4.70 | 0.20 | 63.31±5.33 | 63.55±7.22 | 0.92 |
| 19.62±3.43 | 22.42±2.43 | 0.02^*^ | 21.41±4.24 | 23.51±2.79 | 0.15 |
| 23.70±4.00 | 22.02±2.77 | 0.22 | 25.1±2.70 | 22.21±2.23 | 0.01^*^ |
| 29.95±5.56 | 22.18±2.13 | ＜0.01^**^ | 29.19±5.18 | 25.38±2.60 | 0.03^*^ |
| 32.38±4.84 | 34.84±2.84 | 0.12 | 37.79±4.96 | 36.45±2.65 | 0.51 |
| 39.6±6.87 | 44.81±2.79 | 0.02^*^ | 39.94±5.04 | 45.34±3.00 | ＜0.01^**^ |
|  |  |  |  |  |  |
| 64.47±9.60 | 81.17±6.55 | ＜0.01^**^ | 69.09±6.32 | 83.17±8.57 | ＜0.01^**^ |
| 48.32±8.73 | 45.33±5.08 | 0.30 | 51.80±6.39 | 51.52±7.18 | 0.91 |
| 73.13±5.66 | 75.11±5.98 | 0.40 | 71.65±3.37 | 75.65±6.41 | 0.06 |
| 43.5±6.76 | 41.30±5.96 | 0.39 | 43.64±2.85 | 43.03±4.74 | 0.69 |
| 40.00±6.00 | 41.88±4.45 | 0.37 | 46.18±6.61 | 45.79±2.49 | 0.84 |
|  |  |  |  |  |  |
| 86.64±7.10 | 85.09±8.14 | 0.61 | 89.58±7.86 | 87.72±5.65 | 0.49 |
| 80.34±7.56 | 81.70±4.27 | 0.67 | 85.64±8.26 | 83.77±7.09 | 0.54 |
| 5.70±3.98 | 4.70±2.43 | 0.45 | 5.57±2.70 | 4.81±1.56 | 0.39 |
| 12.53±9.02 | 8.83±3.34 | 0.18 | 11.39±5.20 | 7.46±4.53 | 0.05 |
| 120.93±8.19 | 121.15±8.20 | 0.94 | 119.23±8.39 | 121.08±8.65 | 0.58 |
| 90.10±8.72 | 100.81±3.60 | ＜0.01^**^ | 95.04±10.75 | 100.76±3.32 | 0.08 |
| 96.09±11.28 | 125.12±2.92 | ＜0.01^**^ | 104.47±6.89 | 123.24±2.49 | ＜0.01^**^ |
| 169.09±5.76 | 171.95±2.66 | 0.26 | 170.75±9.44 | 175.23±2.50 | 0.11 |
| 156.80±8.93 | 124.88±2.80 | ＜0.01^**^ | 153.68±9.20 | 129.93±4.97 | ＜0.01^**^ |
