# Supplementary material for: Craniofacial development in patients of Tessier No.0 cleft with a bifid nose using 3D computed tomography
Source: Front Pediatr. 2022 Aug 24;10:979345. doi: 10.3389/fped.2022.979345 (PMC9449123; doi:10.3389/fped.2022.979345)
Supplement: Supplementary file 2 [file Table_2.DOCX]

Supplement Table 1. Definition of landmarks, cephalometric distances and angles.

| Variable | Abbreviation | Definition |
| --- | --- | --- |
| Zygomaticofrontal suture | Z | On the orbital margin of the anterior point of the zygomatic frontal suture |
| Orbitale | Or | The lowest point of the bony inferior orbital margin |
| Superior nasomaxillary suture | SNM | Superior point of suture between nasal bone and maxilla |
| Lateral nasal center point | LNC | Middle point of suture between nasal bone and maxilla |
| Middle point of lateral piriform aperture | MPA | Inferior point of suture between nasal bone and maxilla |
| Alare | Al | lateral piriform aperture at the outermost and lower point |
| Nasion | N | Junction of the frontonasal suture at the most posterior point on the curve at the bridge of the nose |
| Basion | Ba | Most inferior and posterior point on the most anterior margin of the foramen magnum |
| Sella | S | Center of the hypophyseal fossa |
| Anterior nasal spine | ANS | The tip of the median, sharp bony process of the maxilla at the lower margin of the anterior nasal opening |
| Posterior nasal spine | PNS | Most posterior midpoint of the posterior nasal spine of the palatine bone |
| Pogonion | Pog | Most anterior midpoint of the chin on the outline of the mandibular symphysis |
| Spheno-occipital synchondrosis | SO | Most anterior point on the midline of the occipital bone at the spheno-occipital synchondrosis |
| Ethmo-sphenoid | ES | Most superior point of the suture between ethmoid and sphenoid at the midline |
| A point | A | Point of maximum concavity in the midline of the alveolar process of the maxilla |
| B point | B | Point of maximum concavity in the midline of the alveolar process of the mandible |
| Liner measurements |  |  |
|  | ZL-ZR | The distance between the both Zygomaticofrontal suture |
|  | OrL-OrR | The distance between the both Orbitale |
|  | SNML-SNMR | Distance from the superior point of bilateral nasomaxillary suture |
|  | LNCL-LNCR | Distance from the middle point of bilateral nasomaxillary suture |
|  | INML-INMR | Distance from the inferior point of bilateral nasomaxillary suture |
|  | MPAL-MPAR | Distance from the midpoint of the lateral piriform aperture on both sides |
|  | AlL-AlR | The distance between the most lateral and inferior points of the nasal aperture, indicates the nasal base width |
|  | N-Ro | The distance between nasion and rhinion; indicates nasal length |
|  | ANS-PNS | Indicates the length of hard palate |
|  | N-ANS | Indicates the upper anterior facial height |
|  | N-PNS | The distance between Nasion and posterior nasal spine |
|  | N-Ba | The distance between Nasion and Basion, indicates the cranial base length |
|  | S-Ba | The distance between Sella and Basion, indicates the posterior cranial base length |
|  | S-N | The distance between Sella and Nasion, indicates the anterior cranial base length |
|  | S-SO | The distance between Sella and Spheno-occipital Synchondrosis, indicates the cranial base length |
|  | S-ES | The distance between sella and ethmo-sphenoid, indicates the sphenoid endocranial length |
|  | SO-Ba | The distance between Spheno-occipital Synchondrosis and Basion, indicates the length of basilar part of occipital bone |
|  | SO-ES | The distance between Spheno-occipital Synchondrosis and Ethmo-sphenoid, indicates the anteroposterior length of sphenoid |
|  | N-ES | The distance between Nasion and ethmoid-sphenoid, indicates the ethmoid length in anterior cranial fossa |
|  | Ba-ANS | Indicates the midfacial length |
|  | Ba-PNS | The distance between basion and posterior nasal spine, indicates the length of posterior cranial base, and the anteroposterior diameter of airway |
|  | S-PNS | The distance between nasion and posterior nasal spine, indicates the distance of middle cranial base to posterior nasal spine |
|  | ES-PNS | The distance between the suture of ethmoid-sphenoid and posterior nasal spine, indicates the distance of anterior cranial base to posterior nasal spine |
| Angular measurements |  |  |
|  | SNA | Indicates the sagittal position of the maxilla relative to the cranial base |
|  | SNB | Indicates the sagittal position of the mandible relative to the cranial base |
|  | ANB | The relative position of the maxilla to the mandible |
|  | NA-PA | Included angle of extension line of nasion to A and Pogonion to A, indicates facial convexity angle |
|  | N-S-Ba | Indicates the cranial base angle in brain side |
|  | N-S-SO | Indicates the middle and anterior cranial base angle |
|  | S-SO-Ba | Indicates the posterior cranial base angle |
|  | N-SO-Ba | Indicates the cranial base angle in facial side |
|  | Ba-S-ES | Indicates the middle and anterior cranial base angle |
